# Recent Advances in Aptamer-Based Microfluidic Biosensors for the Isolation, Signal Amplification and Detection of Exosomes

**DOI:** 10.3390/s25030848

**Published:** 2025-01-30

**Authors:** Jessica Hu, Dan Gao

**Affiliations:** 1 State Key Laboratory of Chemical Oncogenomics, Tsinghua Shenzhen International Graduate School and Open FIESTA, Tsinghua University, Shenzhen 518055, China; hzs23@mails.tsinghua.edu.cn; 2 Key Laboratory of Metabolomics at Shenzhen, Shenzhen 518055, China

**Keywords:** exosome, microfluidics, aptamer, isolation, signal amplification, detection

## Abstract

Exosomes carry diverse tumor-associated molecular information that can reflect real-time tumor progression, making them a promising tool for liquid biopsy. However, traditional methods for exosome isolation and detection often rely on large, expensive equipment and are time-consuming, limiting their practical applicability in clinical settings. Microfluidic technology offers a versatile platform for exosome analysis, with advantages such as seamless integration, portability and reduced sample volumes. Aptamers, which are single-stranded oligonucleotides with high affinity and specificity for target molecules, have been frequently employed in the development of aptamer-based microfluidics for the isolation, signal amplification, and quantitative detection of exosomes. This review summarizes recent advances in aptamer-based microfluidic strategies for exosome analysis, including (1) strategies for on-chip exosome capture mediated by aptamers combined with nanomaterials or nanointerfaces; (2) aptamer-based on-chip signal amplification techniques, such as enzyme-free hybridization chain reaction (HCR), rolling circle amplification (RCA), and DNA machine-assisted amplification; and (3) various aptamer-assisted detection methods, such as fluorescence, electrochemistry, surface-enhanced Raman scattering (SERS), and magnetism. The limitations and advantages of these methods are also summarized. Finally, future challenges and directions for the clinical analysis of exosomes based on aptamer-based microfluidics are discussed.

## 1. Introduction

Exosomes are extracellular vesicles (EVs) with a typical size range from 50 to 200 nm [1]. Through the inheritance of abundant signaling molecules (e.g., nucleic acids, proteins, and lipids) from donor cells, exosomes facilitate intercellular communication and are associated with tumor progression, immune regulation, and metastasis [2]. Their phospholipid bilayer membrane enables stable detection in various body fluids, including plasma and urine, reflecting real-time tumor loading status and thereby are considered valuable tools for liquid biopsy [3]. Current strategies for exosome detection primarily focus on analyzing membrane proteins, internal proteins, and nucleic acids. To identify these biomarkers, antibodies and aptamers are frequently employed, often in combination with nanomaterials or signal molecules, such as magnetic beads (MBs) and fluorophores, to facilitate downstream analysis.

Aptamers are single-stranded DNA (ssDNA) and RNA isolated from in vitro selective oligonucleotide sequences known as systematic evolution of ligands by exponential enrichment (SELEX) [4]. Aptamers can adopt three-dimensional (3D) conformations, enabling them to bind target molecules with high affinity and specificity. Because of their distinctive properties, including high stability, cost-effectiveness, low immunogenicity, and ease of synthesis and selection, aptamers are increasingly employed as alternatives to antibodies and peptides for binding various small-molecule targets, such as ions, exosomes and tumor markers [5]. In recent years, numerous studies have proposed aptamer-based strategies for the capture and detection of exosomes. For instance, Zhang et al. [6] employed a CD63 aptamer to capture exosomes and found that the quantity of MUC1^+^ exosomes could serve as a marker for identifying cancer patients. In terms of exosome detection, Lei et al. [7] developed an assay to quantify tumor-associated miRNAs by labeling tumor-derived exosomes with allosteric EpCAM and CD63 aptamers, which serve as input units for an orthogonal identity barcode to initiate the miRNA detection process via targeted vesicle fusion. Although these studies highlight the significant versatility of aptamers in meeting various application demands, the suggested designs still have limitations, such as the necessity of large sample volumes, prolonged processing time, and poor efficiency.

In fact, the effective isolation and reliable analysis of exosomes are crucial for their clinical applications. However, traditional exosome isolation and detection methods, such as ultracentrifugation and western blotting, generally necessitate large sample volumes for purification, rely on costly, large-scale equipment, and are often time-consuming. To address these challenges, studies have proposed using microfluidic technology for exosome separation, enrichment, and quantitative detection. These microfluidic devices enable the isolation of highly purified exosomes and the detection of specific exosome subpopulations within small sample volumes of body fluids (range from 1 aL to 1 nL) [8,9,10]. Furthermore, some devices may even enable the simultaneous multiplexed profiling of multiple tumor-specific markers on exosomes [11].

Building on these advantages, recent studies have explored the integration of aptamer-based recognition elements into microfluidic devices for the isolation and detection of cancer-derived exosomes. These aptamer-functionalized microfluidic systems integrate multiple advantages, including reduced reagent and sample consumption, enhanced throughput, minimized contamination, and the elimination of extensive sample preprocessing required in traditional approaches [12]. By providing a well-structured microenvironment for immobilizing recognition probes, such as aptamers, microfluidic platforms enable efficient exosome capture while precise fluid manipulation minimizes non-specific adsorption and enhances target-probe interactions [13]. These unique features have spurred substantial advancements in the development of highly sensitive and specific biosensing platforms for exosome-based cancer diagnostics. This review provides an overview of current improvements in aptamer-based microfluidic biosensors for exosome isolation, signal amplification and detection.

## 2. Aptamer-Based Microfluidics for Exosome Isolation

Exosomes contain a variety of transmembrane proteins (e.g., CD63, CD9, CD81) [14] and cancer-associated biomarkers (e.g., EpCAM, EGFR) [15]. Therefore, isolation can be achieved through immunoaffinity-based capture methods utilizing specific antibodies or aptamers. Aptamers present numerous benefits over antibodies, such as superior chemical stability, lower cost, ease of preparation, and the capacity for structural modifications [16,17]. Notably, aptamer-based elution buffers, including salt solutions, complementary DNA strands, and DNase, are considerably milder than those required for antibody-based methods. The harsh conditions, such as extremely low pH, needed to release vesicles from antibody complexes often compromise vesicle integrity. By contrast, the gentle elution conditions of aptamer-based approaches better preserve exosomal structure and biological activity, ensuring their appropriateness for subsequent downstream analysis [18]. Consequently, numerous aptamer-based exosome isolation techniques have been developed. Many of these methods have been further integrated with microfluidic technology. These on-chip aptamer-based exosome isolation methods can be classified into two categories according to the modification sites of the aptamer: (1) aptamer-nanomaterial-mediated capture and (2) aptamer-nanointerface mediated capture.

### 2.1. Aptamer-Nanomaterial-Mediated Capture

**Figure 1:**
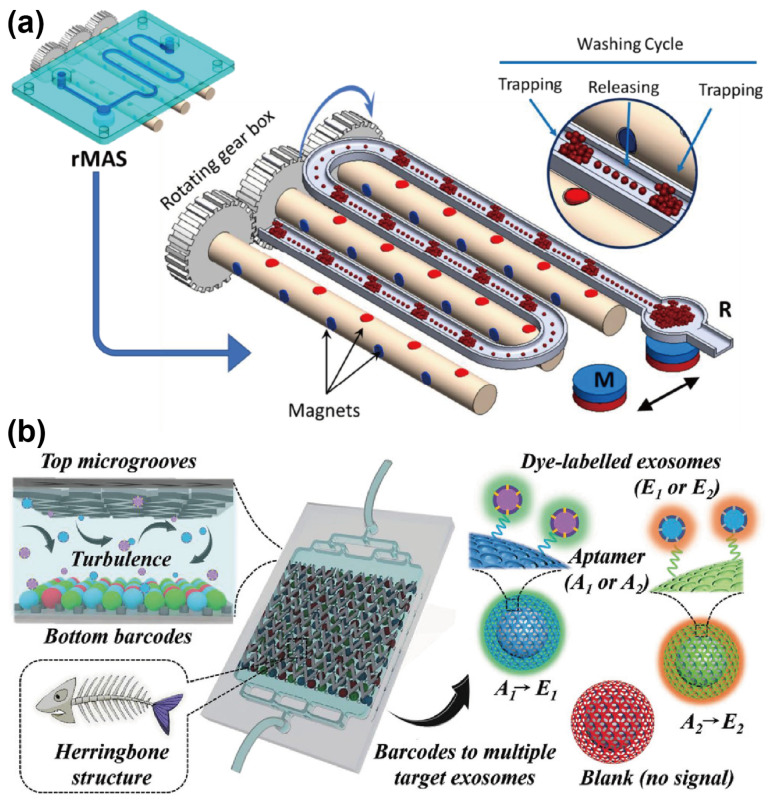
Schematic diagrams illustrating aptamer-nanomaterial-mediated exosome capture. (**a**) Schematic of the rotating magnetic separator. Reprinted from Ref. [20] under Creative Commons Attribution (CC BY) 4.0 License. https://creativecommons.org/licenses/by/4.0/ (accessed on 25 August 2023); (**b**) Schematic of the herringbone microfluidic device with aptamer-modified barcodes for exosome capture and detection. Reprinted with permission from Ref. [22]. Copyright 2022 Wiley-VCH GmbH.

In aptamer-nanomaterial-mediated capture, aptamers are first conjugated to nanomaterials, such as nanospheres, nanoparticles, and nanorods, to facilitate exosome capture. These nanomaterials provide a high-density aptamer immobilization surface, thereby enhancing capture efficiency by increasing the available surface area for aptamer-exosome interaction. In recent years, the integration of immunomagnetic bead (IMB)-based exosome separation techniques with microfluidic platforms have gained significant attention, enabling high-purity, high-throughput, and low-sample-volume isolation of exosomes. Traditionally, these IMB methods applied antibodies as recognition molecules for targeting exosomes. However, such approaches are inherently limited by their inability to achieve the non-destructive release of exosomes, which may compromise their biological integrity and reduce the reliability of downstream analyses. To overcome these limitations, researchers have increasingly adopted aptamers as substitutes for antibodies, developing various strategies for isolating exosomes using aptamer-functionalized MBs. For example, Song et al. [19] developed an MB-based exosome immunoaffinity separation system, marking the first reported use of a CD63 aptamer to target CD63-positive exosomes. This approach successfully isolated 8.37 × 10^7^ exosomes from 10 mL of an MDA-MB-231 and HT29 cell culture medium. The captured exosomes were subsequently eluted using NaCl. Based on this capture principle, Chinnappan et al. [20] introduced a microfluidic magnetic separation system. As shown in Figure 1a, this system utilized carbon-coated magnetic (CCM) beads functionalized with a CEA aptamer to capture breast cancer-derived exosomes specifically. The captured exosomes were efficiently enriched through iterative magnetic capture and release cycles and were ultimately collected at the channel outlet by a fixed magnet for downstream analysis.

Notably, the examples discussed above are all in vitro studies which lack the capability for real-time or dynamic monitoring. To address this limitation, Cong et al. [21] developed a microfluidic device integrated with a mouse model to establish an extracorporeal circulation system for the in vivo separation of PD-L1-positive small extracellular vesicles (sEVs). This system comprises a cell isolation chip and an sEV separation chip. The former facilitates the initial separation of blood cells and sEVs, while the latter employs CD63 aptamer-modified magnetic nanospheres to capture sEVs. The captured sEVs were specifically identified using PD-L1 aptamer. With the proposed system, a 60% separation efficiency for sEVs was achieved directly from in vivo whole blood samples. In addition to MBs, nanomaterials, such as barcodes, can also be functionalized with aptamers to enhance exosome isolation efficiency, especially from low-abundance samples. Chen et al. [22] demonstrated the use of aptamer-functionalized core-shell barcodes (AFCSBs) in a herringbone microfluidic device for specific exosome capture. The barcode shells, with interconnected pores, provide extensive surface area for aptamer binding, while their integration into herringbone grooves enhances exosome interaction by increasing resistance to turbulent flow (Figure 1b). Experimental results demonstrated that this design could achieve an exosome capture efficiency of approximately 60% directly from peripheral blood.

### 2.2. Aptamer-Nanointerface-Mediated Capture

**Figure 2:**
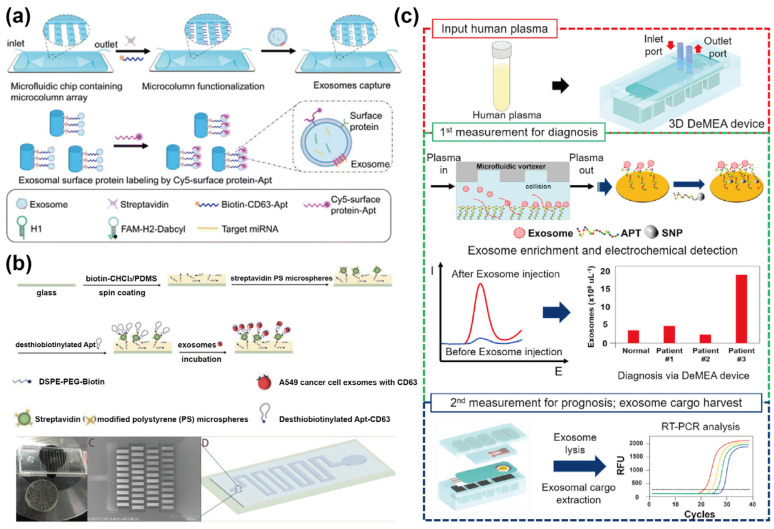
Schematic illustration of aptamer–nanointerface–mediated exosome capture. (**a**) Illustration of exosome capture and simultaneous detection of exosomal proteins and miRNAs on a microfluidic chip. Adapted from Ref. [23]; (**b**) Schematic of a microfluidic chip for exosome isolation using multi–target aptamers. Reprinted from Ref. [25] under Creative Commons Attribution (CC BY) 4.0 License. https://creativecommons.org/licenses/by/4.0/ (accessed on 18 April 2022); (**c**) Diagrams of aptamer–functionalized electrochemical microfluidic sensors. Reprinted with permission from Ref. [28]. Copyright 2020 Elsevier B.V.

In addition to aptamer-functionalized nanomaterials, aptamers can also be directly immobilized onto the microstructured surfaces of the microfluidic chip, thereby facilitating exosome capture. For instance, our group [23] designed a ship-shaped microfluidic device to capture and detect exosomes derived from non-small cell lung cancer. As illustrated in Figure 2a, this device incorporated circular micropillar arrays, which enhance the surface-to-volume ratio. By modifying these PDMS micropillars with CD63 aptamers, the device achieved specific exosome capture while simultaneously allowing in situ detection of exosomal miRNAs and surface proteins. To further improve capture efficiency, our group [24] replaced the circular micropillar arrays with Y-shaped array channels in a similar ship-shaped microfluidic device. Experimental results showed that Y-shaped channels functionalized with CD63 aptamers, which have a higher surface-to-volume ratio than circular micropillars, could enhance exosome capture efficiency to approximately 65%. Notably, the aforementioned methods employed single-target aptamers to capture specific cancerous exosomes. However, single-target capture may exhibit insufficient specificity due to the limited specificity of exosomal surface proteins, which can compromise the accuracy of exosome isolation. Therefore, using multi-target aptamers for exosome isolation can significantly enhance capture specificity. For example, Zhou et al. [25] proposed a method utilizing CD63 and PTK7 aptamers to isolate CD63 and PTK7-positive exosomes from cell culture supernatants directly. The device incorporated a microfilter at the inlet to remove impurities from the supernatant while the aptamers were immobilized on the glass substrate of the microchannel through a biotin-avidin-desthiobiotin bond (Figure 2b). This approach enabled the high-specificity isolation of exosomes from a heterogeneous population of EVs. Experimental results demonstrated that the device was capable of isolating approximately 10^7^–10^8^ particles/mL of CD63 and PTK7-positive exosomes within 20 min.

Moreover, using an electrochemical biosensor as a powerful tool for exosome detection, offers advantages such as high sensitivity and specificity [26]. However, repeated modification steps can decrease electrical conductivity, and the use of various biological reagents may compromise the stability of the sensing surface [27]. As a result, electrochemical sensors are generally limited in their use as exosome detection tools. To address these challenges, Kashefi-Kheyrabadi et al. [28] proposed a nanocomposite comprising MoS_2_ nanosheets, graphene nanoplatelets, and chitosan to enhance the conductivity of the sensing surface, improve the immobilization efficiency of aptamers, and increase surface biocompatibility (Figure 2c). The device achieved specific capture of cancerous exosomes by functionalizing the sensing surface with EpCAM aptamers. To further enhance performance, a geometrically activated surface interaction microfluidic channel was integrated, generating micro-vortices that increased the collision frequency between exosomes and the sensing surface. Experimental results showed that this design enabled the chip to capture high-purity exosome samples from 10 μL samples.

Although aptamers exhibit high specificity and the ability to preserve the biological activity of exosomes through simple integration with microfluidics, aptamers are susceptible to nuclease breakdown and exhibit reduced affinity in complex biological fluids, influenced by factors such as pH, buffer composition, salt concentration, as well as DNA sequence and length [17,29,30]. Additionally, the availability of high-affinity aptamers targeting exosomal surface proteins still needs to be improved. Therefore, future research is needed to improve the affinity of aptamers and optimize modification strategies, buffer conditions, and microchannel designs to maintain aptamer stability and structural integrity within microfluidic systems. These advancements will enhance the binding affinity of aptamers, strengthen interactions between aptamers and exosomes, and ultimately improve the efficiency of exosome capture.

## 3. Aptamer-Based On-Chip Signal Amplification Methods

Recent advancements in nucleic acid technologies have highlighted the dual functionality of aptamers. These molecules not only enable the specific capture of exosomes but introduce innovative approaches for signal transduction and amplification. Common nucleic acid amplification methods include enzyme-free amplification techniques (e.g., catalyzed hairpin assembly, hybridization chain reaction, catalytic hairpin assembly, and toehold-mediated strand displacement), enzyme-based amplification methods (e.g., rolling circle amplification, loop-mediated isothermal amplification, and recombinase polymerase amplification), DNA machine-assisted amplification, and CRISPR/Cas system-mediated signal amplification. In the context of on-chip exosome detection, aptamer-assisted signal amplification strategies predominantly involve enzyme-free hybridization chain reaction (HCR), rolling circle amplification (RCA), and DNA machine-assisted amplification. This section provides an overview of these three aptamer-based signal amplification strategies for exosome analysis.

### 3.1. Enzyme-Free HCR-Based Amplification Method

**Figure 3:**
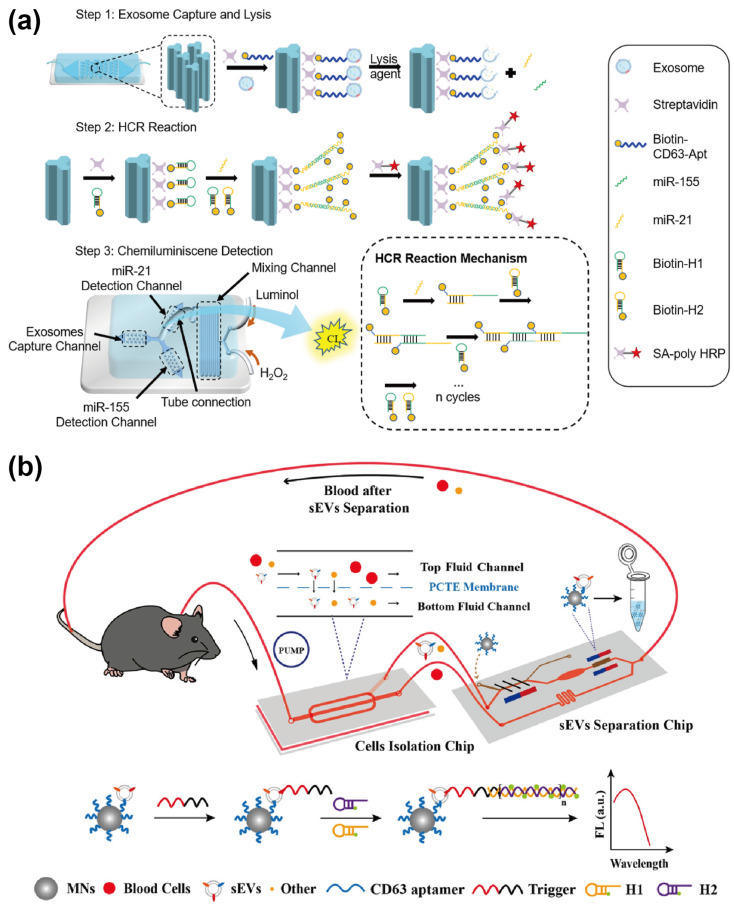
Schematic illustration of aptamer-mediated HCR amplification method. (**a**) Schematic diagram of the microfluidic chemiluminescence sensors for the HCR process and chemiluminescence detection of the exosomal miRNA. Reprinted with permission from Ref. [24]. Copyright 2024 Elsevier B.V. (**b**) Schematic diagram of the aptamer recognition and HCR process of PD-L1^+^ sEVs. Reprinted with permission from Ref. [21]. Copyright 2024 American Chemical Society.

The hybridization chain reaction (HCR) is a well-established enzyme-free and isothermal amplification strategy that relies on three essential components: two hairpin structures (H1 and H2) and an initiator strand. The initiator strand triggers sequential strand displacement and hybridization reactions between the hairpins, forming an extended double-stranded DNA (dsDNA) structure [31]. By leveraging aptamers, the enzyme-free HCR amplification method can be adapted for exosome detection. With their ability to specifically bind to the exosome surface, aptamers enable the integration of the HCR system, which converts target exosome information into detectable signals. For instance, our group [24] developed a microfluidic chip featuring a Y-shaped array to detect miRNAs derived from breast cancer exosomes. As described previously, exosomes were specifically identified using CD63 aptamers, and the identified exosomes were subsequently lysed to release miRNA. As shown in Figure 3a, the released miRNAs triggered an HCR reaction via biotin-modified hairpin probes (H1 and H2). This reaction subsequently activated the chemiluminescence process through streptavidin-biotin interactions, enabling the quantitative detection of exosomal miRNAs, including miR-21 and miR-155. Using this method, the platform achieved a limit of detection (LOD) of 0.49 fM for exosome analysis. Beyond directly employing miRNAs as initiators for HCR, aptamers can also function as mediators to facilitate signal transduction. For example, Cong et al. [21] designed a platform for detecting PD-L1^+^ sEVs (Figure 3b). This approach employed magnetic nanosphere (MN)-functionalized CD63 aptamers to capture exosomes specifically. A trigger sequence containing a PD-L1 aptamer was then employed to recognize PD-L1 on the exosomes and initiate HCR-mediated fluorescence amplification, enabling quantitative detection of PD-L1. This platform achieved an LOD of 3.2 × 10^3^ particles/μL, underscoring its potential for highly sensitive detection of exosomal proteins.

Although the enzyme-free HCR method can provide a cost-effective, durable, and highly sensitive approach for detecting low-abundance exosomes, its amplification efficiency remains relatively limited. Additionally, this method often involves long reaction times and stringent sequence design requirements, as potential circuit leakage can increase background signals and reduce detection accuracy.

### 3.2. RCA-Based Method

Rolling circle amplification (RCA) is an isothermal nucleic acid amplification technique extensively employed in various biosensing applications. RCA utilizes a circular DNA template and a complementary primer to initiate a continuous DNA synthesis process catalyzed by a highly processive DNA polymerase. Unlike conventional amplification methods, RCA operates under constant temperature conditions, generating long, repetitive ssDNA sequences that serve as scaffolds for signal generation or subsequent amplification processes [32,33]. When integrated with aptamers capable of specifically recognizing exosomes, RCA provides a robust and highly sensitive strategy for exosome detection, offering significant potential in diagnostic and analytical applications.

**Figure 4:**
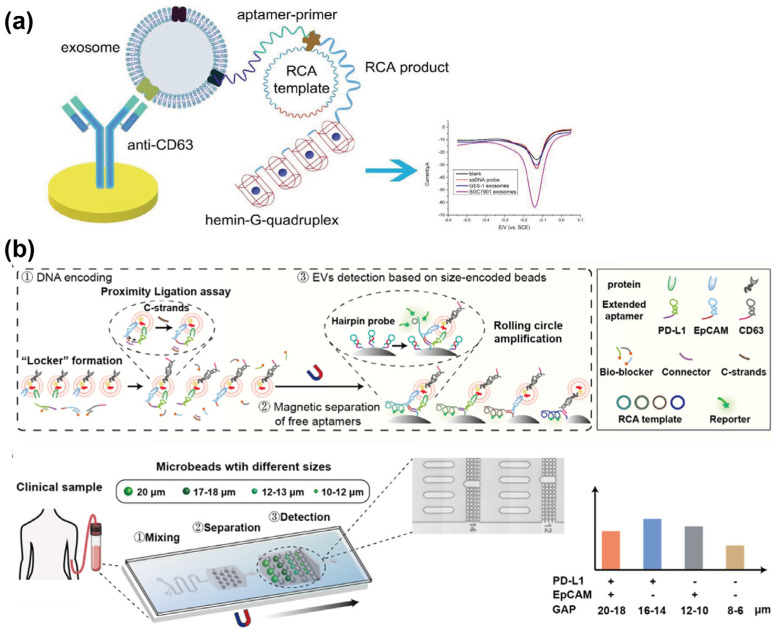
Schematic representation of aptamer–assisted RCA signal amplification. (**a**) Principle of the electrochemical aptasensor for detecting gastric cancer–derived exosomes. Reprinted with permission from Ref. [34]. Copyright 2019 Wiley–VCH Verlag GmbH & Co. KGaA, Weinheim; (**b**) Illustration of the size–coded affinity matrix microfluidic chip for multiplexed EV phenotyping. Reprinted with permission from Ref. [37]. Copyright 2022 American Chemical Society.

Using the RCA method, Huang et al. [34] developed a label-free electrochemical aptasensor for the specific detection of gastric cancer-derived exosomes. As depicted in Figure 4a, the platform captures exosomes via an anti-CD63 antibody-functionalized electrode. Following exosome capture, the MUC1 aptamer acts as both a recognition probe and a primer to initiate the RCA process. This process facilitates the formation of the hemin/G-quadruplex system, which catalyzes the reduction of H_2_O_2_, generating measurable electrochemical signals. The proposed aptasensor demonstrated high selectivity and sensitivity for gastric cancer exosomes, achieving an LOD of 9.54 × 10^2^ particles/mL. Coupling with another sensing method, Zhao et al. [35] developed a surface-enhanced Raman scattering (SERS) sensor for the detection of exosomal miRNA, utilizing a combination of RCA and tyramine signal amplification (TSA). In this strategy, exosomes are selectively captured using CD63 aptamer-functionalized MBs. The miRNA released from the exosomes then activates the RCA process, subsequently triggering the SERS detection process.

However, previously discussed methods focus primarily on the specific recognition of a single biomarker, neglecting the inherent heterogeneity of EVs, which can compromise the accuracy of EV identification. To address this limitation, Wu et al. [34] introduced a size-coded affinity matrix microfluidic chip designed for multiplexed EV phenotyping. As shown in Figure 4b, this aptasensor employs microbeads of varying sizes, each functionalized with hairpin probes that contain CD63, EpCAM and PD-L1 aptamers specific to a range of EV surface biomarkers. Subsequently, in situ RCA is used to amplify signals from aptamers bound to the corresponding EV biomarkers on each microbead population. To minimize background interference from aptamers that do not specifically bind to their respective EV targets, biotinylated “Bioblocker” probes are annealed with the aptamers, forming “Locker” molecules. These molecules retain non-specifically bound aptamers, facilitating their efficient removal. By utilizing microbeads of varying sizes conjugated with hairpin probes, the proposed approach enabled the analysis of PD-L1 and EpCAM expression patterns in EV samples derived from A375, U251, MCF-7, and Jurkat cell lines. Experimental results demonstrated that the proposed approach enables the rapid and simultaneous detection of six distinct EV phenotypes, with an LOD as low as 9.54 × 10^2^ particles/mL. The exceptional sensitivity of this method, combined with its ability to quantify multiphenotypic EVs, highlights its significant potential for enhancing the precise detection of EVs associated with specific disease phenotypes.

RCA provides high sensitivity by generating substantial amounts of ssDNA, making it particularly suitable for detecting low-abundance targets. Its isothermal nature streamlines the amplification process and offers robust specificity when paired with appropriately designed primers. However, non-specific amplification and the requirement for precise primer design present significant challenges, which can undermine the accuracy of the method [36].

### 3.3. DNA Machine-Assisted Method

DNA walkers, as a class of molecular nanomachines, have emerged as powerful tools in biosensing due to their ability to achieve signal amplification through precise, stepwise movement along a designated track. Typically comprising a walking strand, a track, and a driving motor, these systems can be activated by specific stimuli to initiate the walking process [38]. When integrated with aptamers, DNA walkers exhibit enhanced sensitivity and selectivity, particularly in detecting low-abundance biomarkers such as exosomes. For example, Feng et al. [39] reported a DNA walker-based electrochemiluminescence (ECL) aptasensor for the detection of lung cancer-derived exosomes. The system utilized a RuSi nanoparticle-modified electrode surface. An anchor DNA was employed as the track, along with a dsDNA formed by partial hybridization between a swing arm and a CD63 aptamer. Upon the addition of exosomes, the CD63 aptamer is specifically bound to the CD63 protein on the exosome surface, activating the continuous DNA walking process with an endonuclease. This interaction resulted in the accumulation of significant amounts of ssDNA on the electrode, facilitating the downstream ECL signal detection process. By measuring the ECL signal, the system achieved a high correlation coefficient (R^2^) of 0.997 and enabled the quantitative detection of exosomes, with an LOD of 60 particles/µL.

**Figure 5:**
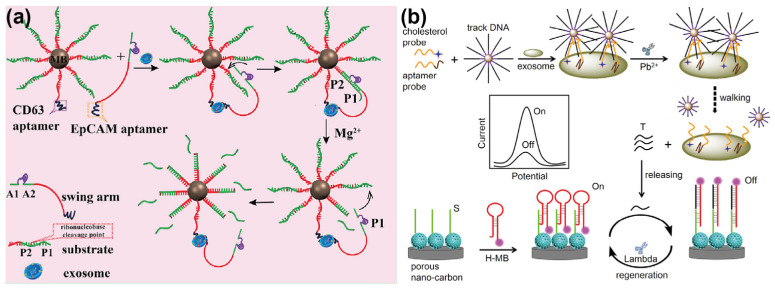
Schematic illustration of aptamer–based DNA machine–assisted signal amplification. (**a**) Illustration of the 3D DNA walker integrated with an Exo III–assisted electrochemical ratiometric assay. Reprinted with permission from Ref. [40]. Copyright 2019 American Chemical Society; (**b**) Depiction of the operational principle of an electrochemical biosensor leveraging a dual–recognition proximity–induced DNA walker. Reprinted with permission from Ref. [41]. Copyright 2021 Elsevier B.V.

In the aforementioned study, the signal amplification mediated by the DNA walker was conducted directly on a two-dimensional (2D) electrode surface. To further enhance the amplification efficiency, modified tracks on the nanoparticles (a 3D walker) can be employed to initiate the DNA walking process as this approach increases the coverage of the walking strand, thereby improving the overall efficiency of the walking process. For instance, Zhao et al. [40] proposed a 3D DNA walker-based strategy for the specific recognition and signal amplification of breast cancer-derived exosomes (Figure 5a). In this system, a CD63 aptamer and an EpCAM aptamer were utilized as the capture and recognition probes, respectively. Substrate strands modified on the MBs (containing a CD63 aptamer) served as the tracks for the 3D DNA walking machine. Signal detection was achieved using an Exo III-assisted electrochemical ratiometric biosensor. By employing dual-probe recognition, the system demonstrated high specificity and low background detection of exosomes in various biological environments, achieving an LOD as low as 1.3 × 10^4^ particles/mL with R^2^ of 0.9908. It is worth noting that DNA walkers utilizing only a single type of DNA walking strand on nanoparticles may fall short of the accuracy requirements for clinical applications in exosome detection. To address this limitation, Guo et al. [41] developed an electrochemical biosensor based on dual-recognition proximity-induced activation of a DNA walker for exosome detection. As illustrated in Figure 5b, this strategy involves the design of two proximity probes: one featuring a Pb^2+^-dependent DNAzyme tail sequence and the other comprising a recognition probe formed by cholesterol and an aptamer. These probes enable both the recognition of exosomes and the activation of the DNA walker, resulting in the release of numerous intermediate DNA strands. These strands subsequently participate in signal transduction on the electrode surface, facilitating the quantitative detection of exosomes. Experimental results demonstrated that this signal amplification strategy achieved an LOD as low as 1.6 × 10^4^ particles/mL, with low background, high specificity, and excellent reproducibility in exosome quantification.

In summary, the combination of aptamers’ high specificity for exosome recognition with the programmability and versatility of DNA walkers offers a highly sensitive and specific approach for exosome detection. However, aptamer-based DNA walkers also encounter several challenges. Their walking efficiency can be affected by steric hindrance or suboptimal hybridization kinetics, particularly in complex biological environments. Additionally, DNA-based systems are susceptible to enzymatic degradation and non-specific interactions, which may compromise detection accuracy and reproducibility [42]. These limitations require further research to fully realize the potential of aptamer-based DNA walkers in clinical applications.

## 4. Aptamer-Based Microfluidics for Exosome Detection

Aptamers have been extensively developed for the sensitive detection of exosomes, exploiting their versatile structural design and affinity regulation to mediate signal amplification and transduction [12]. In combination with microfluidics, which offers advantages such as low reagent consumption, higher throughput, and easy integration, many aptamer-based microfluidic devices have been developed to detect tumor-derived exosomes. These platforms integrate various signal detection methods, including fluorescence, electrochemistry, surface-enhanced Raman scattering (SERS), and magnetism, thereby providing high-throughput, sensitive, and specific strategies for exosome detection.

### 4.1. Fluorescence-Based Aptasensor

Fluorescence is a method used for the quantitative analysis of exosomes by converting the target signal into fluorescence intensity. In fluorescence-based aptasensors, aptamers are typically labeled with fluorophores or quenchers, which convert non-fluorescent targets into fluorescent signal molecules. The advantages of this approach include the simplicity of labeling, as well as the inherent capability for real-time detection and multi-signal output. These features have made fluorescence-based aptasensors widely applicable in microfluidic-based exosome detection systems. For instance, Dong et al. [43] developed an ExoID-Chip for the efficient isolation and highly sensitive detection of breast cancer-derived exosomes. In this device, exosomes are first isolated from the cell culture medium through a double filtration process, after which they are selectively recognized by an excess of CD63 aptamers on a photonic crystal (PC) nanostructure. The unbound aptamers are subsequently filtered through a membrane and bind to CD63 immobilized on the PC membrane. Streptavidin-horseradish peroxidase (SA-HRP) conjugates then interact with the biotinylated aptamers, and upon the addition of H_2_O_2_ and peroxidase substrates, a fluorescent product, resorufin, is generated. Fluorescence images are acquired using a fluorescence microscope. This device requires only 20 μL of sample and achieves an LOD of 8.9 × 10^3^ particles/mL.

**Figure 6:**
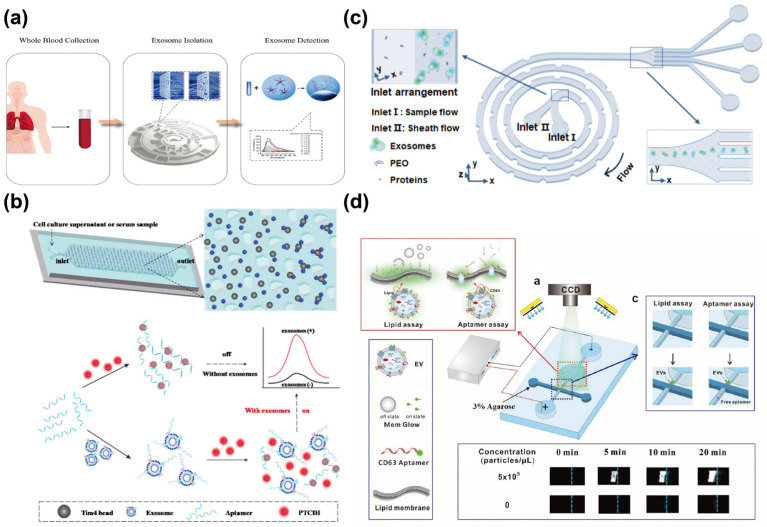
Schematic diagrams of microfluidic fluorescence–based aptasensors. (**a**) The principle of the Exo–CMDS and Exo–AFS process. Reprinted from [44] under Creative Commons Attribution (CC BY) 4.0 License. https://creativecommons.org/licenses/by/4.0/ (accessed on 15 October 2022); (**b**) Schematic of the isolation process and the PTCDI–aptamer signal switch strategy. Reprinted with permission from Ref. [45]. Copyright 2022 American Chemical Society; (**c**) Schematic illustration of DEIC chip for the isolation and detection of exosome. Reprinted with permission from Ref. [46]. Copyright 2023 American Chemical Society; (**d**) Schematic of the lipid assay and the aptamer assay. Reprinted with permission from Ref. [47]. Copyright 2021 Elsevier B.V.

Using similar detection components, Zhao et al. [44] utilized PD-L1 aptamer-functionalized MBs to capture lung cancer-derived exosomes (Exo-AFS). As shown in Figure 6a, the system employs HRP-catalyzed reactions between H_2_O_2_ and Amplex Red, generating a fluorescent signal. Aptamer-based one-step rapid detection is achieved by competitively releasing aptamer-complementary probes through binding with exosomal membrane proteins. Integrated with an upstream automated centrifugal microfluidic disc system (Exo-CMDS), which facilitates a one-step process for whole blood injection and exosome collection, this device enables exosome detection with an LOD of 1.58 × 10^5^ particles/mL within 8 min and achieves a lung cancer diagnostic accuracy of 91%. Using different chip structures and fluorescent probes, Zheng et al. [45] developed a microfluidic platform incorporating drop-shaped micropillars for the specific capture of exosomes, combined with a PTCDI-aptamer signal-switching strategy for quantitative exosome analysis. In this platform, Ca^2+^-dependent Tim4-modified MBs are used to capture exosomes, enabling their easy release using ion chelators (Figure 6b). A cationic perylene fluorescent probe, PTCDI, subsequently self-assembles onto the aptamer, where the phosphate anions in the DNA backbone quench their fluorescence. Upon the binding of exosomes to the aptamer, these electrostatic interactions are disrupted, leading to the restoration of fluorescence. This strategy enabled the detection of surface proteins (e.g., CD63, EpCAM, PSMA, and Nucleolin) on exosomes from various cancer cell lines, including HepG2, MCF-7, HeLa, HEK-293T, and L-02 cells. Experimental results demonstrated that this signal probe could distinguish subtle changes in protein expression levels on exosomal surfaces from different cell lines. Using the CD63 aptamer to detect HepG2-derived exosomes, an LOD as low as 8.69 × 10^3^ particles/mL was achieved.

The high heterogeneity of exosomes constitutes a significant challenge for their detection in clinical applications. As highlighted in the aforementioned example, the use of a single aptamer in a single assay for exosome detection can lead to insufficient specificity. In this regard, Bai et al. [46] proposed using a Dean-flow-coupled elasto-inertial microfluidic chip (DEIC) to isolate high-purity exosomes from complex samples (Figure 6c). This approach allows for the simultaneous detection of heterogeneous surface marker expression (EpCAM and PD-L1) on individual exosomes in serum and cell culture medium, using FAM-labeled EpCAM aptamers and AMCA-labeled PD-L1 aptamers. Linear discriminant analysis (LDA) showed that this combination of markers achieved 100% classification accuracy for liver and esophageal cancers. Fluorescent quantification strategies commonly rely on homogeneous EV samples, where protein expression levels are assumed to be similar across individual exosomes. However, for heterogeneous exosome samples, such strategies struggle to distinguish whether changes in protein expression arise from variations in vesicle size or quantity, or from differences in protein expression per vesicle. To address this challenge, Ren et al. [47] developed an electrophoresis-based microfluidic platform for exosome quantification and normalization using phospholipid bilayer staining (Figure 6d). By leveraging the electromigration behavior of EVs in a direct current electric field, the platform enriched EVs at microchannel intersections (lipid assay), enhancing the fluorescence signal by over 400-fold within 20 min. FAM-labeled CD63 aptamers were employed to evaluate protein expression in plasma-derived EVs (aptamer assay). Compared with nanoparticle tracking analysis (NTA), the results showed that normalizing EV quantities revealed significant variations in CD63 expression across plasma samples. This approach highlights the lipid assay’s ability to mitigate deviations caused by protein heterogeneity in different EV samples, enabling more accurate and reliable analyses.

In summary, recent studies have leveraged the ease of aptamer modification to develop various fluorescence-based aptasensors by directly or indirectly conjugating fluorophores to aptamers. When integrated with microfluidic techniques or nanomaterials, these aptasensors enable sensitive detection with minimal sample consumption. To address the challenges associated with exosome heterogeneity, strategies such as multi-target aptamers or dual-mode detection have been employed to improve specificity and accuracy. However, there are still limitations that need to be improved, including lengthy testing times and dependence on external fluorescence detection equipment. These challenges underscore the need for further research and optimization.

### 4.2. Electrochemical-Based Aptasensor

**Figure 7:**
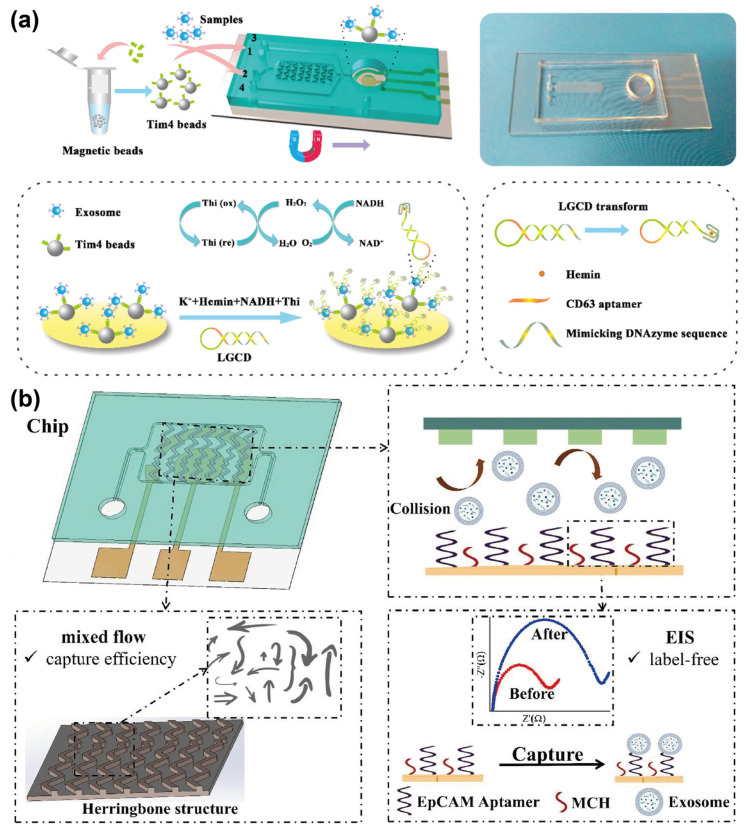
Schematic diagrams of microfluidic electrochemical–based aptasensors. (**a**) Schematic of the ExoPCD–chip and the sensing process. Reprinted with permission from Ref. [49]. Copyright 2018 American Chemical Society; (**b**) Schematic of integrated microelectrode system with a herringbone–structured microfluidic chip. Reprinted with permission from Ref. [51]. Copyright 2024 Elsevier B.V.

Electrochemical-based sensors are widely employed in exosome detection for their high sensitivity, rapid response, and potential for miniaturization. The easily engineered nature of aptamers facilitates their easy modification onto electrodes, where they can serve both as capture probes and signal probes, emitting a signal directly upon analyte binding. By integrating microfluidics with the above detection methods, there is a promising potential to further enhance analytical performance by reducing reagent consumption, streamlining procedures, and improving portability. For instance, Zhou et al. [48] developed an electrochemical aptasensor for the quantitative detection of exosomes. This device incorporated a CD63 aptamer-modified electrode into a microfluidic chip, enabling low-volume sample analysis. In the presence of exosomes, their binding to the aptamer triggered the displacement of the antisense strand, leading to a decrease in the redox signal. Without requiring washing or labeling steps, this platform achieved an LOD as low as 1 × 10^6^ particles/mL, significantly simplifying the exosome detection process. However, the potential non-specific capture of exosomes may reduce the sensitivity of the assay. To fulfill the needs for portable and sensitive exosome analysis, Xu et al. [49] designed a microfluidic platform integrating exosome isolation and in situ electrochemical detection (ExoPCD-chip) for serum analysis. As illustrated in Figure 7a, the platform employed Y-shaped micropillars to enhance capture efficiency and used Tim4-modified MBs to capture exosomes expressing phosphatidylserine in a label-free manner. Subsequently, exosomes were detected using an LGCD probe containing a CD63 aptamer and a mimicking DNAzyme sequence. Binding between exosomes and the LGCD probe induced the formation of a G-quadruplex, which acted as an NADH oxidase and HRP-mimicking DNAzyme to catalyze H_2_O_2_ generation, thereby amplifying the signal. This method eliminates the need for nucleic acid modifications, complex immobilization processes, and additional signal amplification steps. Experimental results demonstrated that this chip further reduced the LOD to 4.39 × 10^3^ particles/mL using only 30 µL of sample, offering a highly efficient and simplified approach to exosome detection.

The mass transfer efficiency at the electrode surface plays a crucial role in determining the electrochemical reaction efficiency. Integrating microfluidics with a nanoscale sensing interface has been demonstrated to enhance mass transport and improve conductivity [50]. In this regard, Li et al. [51] developed an electrochemical aptasensor incorporating a herringbone microfluidic chip for the detection of lung cancer-derived exosomes. The herringbone structure at the sensing interface promotes vertical fluid flow, facilitating the specific binding between exosomes and the aptamers immobilized on the electrode surface (Figure 7b). Upon aptamer-exosome binding, a biomolecular layer forms, impeding electron transfer and increasing electrochemical impedance. By analyzing the resulting electrical signal changes, this system achieved an LOD of 1.4 × 10^4^ particles/mL for label-free tumor exosome detection. In addition to the mass transfer issue, biological samples and multiple modification steps may also weaken the stability and conductivity of the sensing surface, thereby compromising the stability and sensitivity of the sensor. These limitations were addressed in the aforementioned study [28], in which an electrochemical aptasensor (DeMEA) was developed to detect cancerous exosomes from the plasma samples of breast cancer patients. In this system, the EpCAM aptamer functions both as a capture and a signal probe. Once captured, the exosomes bind with redox probe-labeled EpCAM aptamers, leading to electro-oxidation of the redox probe. By measuring changes in the electrochemical signal, the quantity of exosomes in plasma can be quantified. The device demonstrated an LOD as low as 17 particles/μL, and the quantified exosome samples can be further subjected to downstream analysis.

In conclusion, while electrochemical-based aptasensors offer advantages such as rapid response and high sensitivity, their effectiveness in exosome detection is often constrained by the mass transfer efficiency at the sensing surface. Recent advancements in microfluidic-based methods have addressed this issue by incorporating microstructures within chips to improve mass transfer efficiency. However, the stability of the sensing surface and the challenge of biofouling continue to hinder detection accuracy. Although various strategies have been proposed to address these limitations, achieving high sensitivity and stable performance remains challenging.

### 4.3. SERS-Based Aptasensor

Surface-enhanced Raman scattering (SERS) is a highly sensitive analytical technique that significantly amplifies the Raman scattering signals of molecules, enabling the detection of biological and chemical substances with exceptional precision. The sensitivity of SERS arises from the narrow spectral width of Raman peaks, which are typically 10–100 times narrower than fluorescence peaks, providing a distinct molecular fingerprint for individual molecules [52,53]. Integrating aptamers with SERS enables the detection of analytes at extremely low concentrations (picomolar to femtomolar) by leveraging the amplification of Raman signals through metallic nanostructures, enhancing both sensitivity and specificity in complex samples [54].

**Figure 8:**
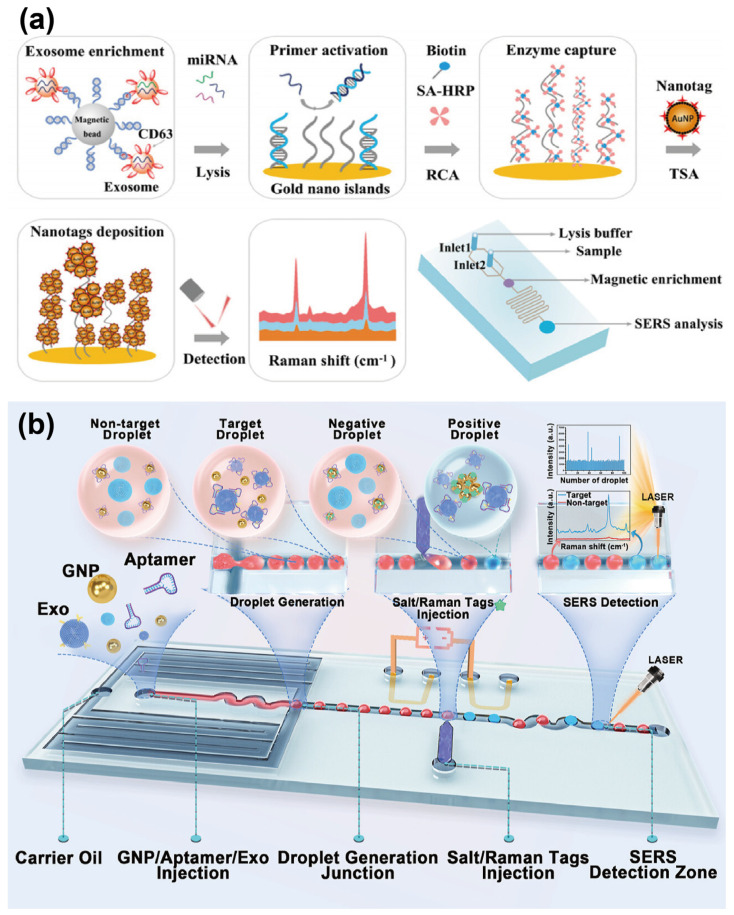
Schematic diagrams of microfluidic SERS–based aptasensors. (**a**) Schematic of microfluidic SERS sensor for the detection of exosomal miRNA with cascade amplification. Reprinted with permission from Ref. [35]. Copyright 2021 Elsevier B.V. (**b**) Schematic of the SERS–based droplet microfluidic platform for detecting HER2-positive exosomes. Reprinted with permission from Ref. [57]. Copyright 2024 American Chemical Society.

For instance, Zhang et al. [55] constructed a dual-aptamer-assisted ratiometric SERS biosensor for the detection of breast cancer-derived exosomes in diluted serum samples. The sensor utilized a substrate composed of regularly arranged Au@Ag nanoparticles/graphene oxide (Au@Ag NPs/GO), with 4-nitrothiophenol (4-NTP) molecules serving as an internal standard. The substrate surface was functionalized with a V-shaped dsDNA formed by a rhodamine X-modified EpCAM aptamer and a HER2 aptamer. Upon specific binding of exosomes to the V-shaped DNA, the SERS signal of rhodamine X decreased, while the signal of 4-NTP remained stable. Experimental results demonstrated that the biosensor achieved an LOD as low as 1.5 × 10^2^ particles/mL without nucleic acid amplification. However, the aforementioned detection process requires a relatively large sample volume and necessitates prior exosome isolation and purification, which can result in poor recovery and potential contamination, thereby complicating the detection process. To address these challenges and streamline the operational workflow, Zhao et al. [35] proposed an integrated microfluidic approach for on-chip enrichment of exosomes coupled with SERS-based detection of exosomal miRNA via RCA cascaded with TSA (Figure 8a). As mentioned, after selectively capturing exosomes using CD63 aptamer-functionalized MBs, miRNA from the exosomes activates primers immobilized on the substrate surface, initiating the RCA process. This, in turn, recruits SA-HRP to catalyze the deposition and amplification of tyramine-labeled SERS nanotags. Through this cascaded signal amplification strategy, the biosensor achieved an extremely low LOD for breast cancer exosomal miRNA down to 1 pmol/L, enabling ultra-sensitive detection of low-abundance targets.

Although the aforementioned examples facilitate the molecular analysis of exosomes, single-function biosensors often suffer from limited versatility and accuracy, hindering their practical adoption. To enhance the clinical applicability of the sensor, Hao et al. [56] proposed an acoustofluidics-assisted bimodal sensing platform capable of performing both immunofluorescent and SERS detection. This platform utilizes surface acoustic waves to aggregate exosomes at desired locations, enabling multiple sensing modes. For the immunofluorescent assay, CD63 aptamer-functionalized silica nanoparticles are used to capture exosomes. Upon applying the acoustic wave, the captured exosomes are concentrated at the center of a glass capillary, thereby enhancing the fluorescent signal. For the SERS assay, the inner wall of the glass capillary is modified with plasmonic Ag nanoparticle-deposited ZnO nanorod arrays, allowing the captured exosomes to be focused on the periphery of the microchannel for SERS detection. Experimental results demonstrated that the platform could achieve LODs of approximately 1.3 × 10^3^ particles/μL for immunofluorescent assays and 20 particles/μL for SERS assays using only 0.5 μL of human plasma-derived exosome samples, enabling rapid detection in both modalities. Notably, the studies mentioned above all require cumbersome off-chip modification steps for SERS nanotags and probes. These additional procedures result in significant reagent consumption and labor-intensive processes, making them unsuitable for high-throughput analysis and point-of-care testing (POCT). To address these limitations and streamline the workflow, Ho et al. [57] introduced a droplet microfluidic platform integrated with a SERS-based aptasensor for the detection of breast cancer-derived exosomes in plasma samples. As shown in Figure 8b, in the presence of exosomes, HER2 aptamers, which are initially bound to gold nanoparticles (GNPs), detach due to stronger affinity with the exosomes. This detachment induces GNP aggregation under high-salt conditions, resulting in SERS signal enhancement through hotspot generation. By monitoring changes in the SERS signal, the platform achieves “sample-to-result” exosome detection with an LOD of 4.5 log_10_ particles/mL within 5 min. This approach significantly simplifies the workflow, facilitating rapid screening and analysis of numerous samples for clinical applications.

In summary, microfluidic-based SERS aptasensors have exhibited remarkable potential for ultra-sensitive exosome detection. These platforms leverage diverse amplification strategies, nanoparticle integration, and external field applications to improve detection throughput and specificity. However, their clinical application still faces significant challenges. For instance, SERS signals are prone to interference from background noise in complex biological samples and the detection of biomarkers at extremely low abundance. Moreover, integrating SERS substrates within microfluidic channels remains a critical challenge, as it directly affects both the spatial uniformity of the substrate and the reproducibility of detection signals [58]. Addressing these limitations through advanced strategies to reduce background interference, enhance signal uniformity, and improve reproducibility is essential for promoting the clinical applicability of SERS-based aptasensors.

### 4.4. Other Aptasensors

**Figure 9:**
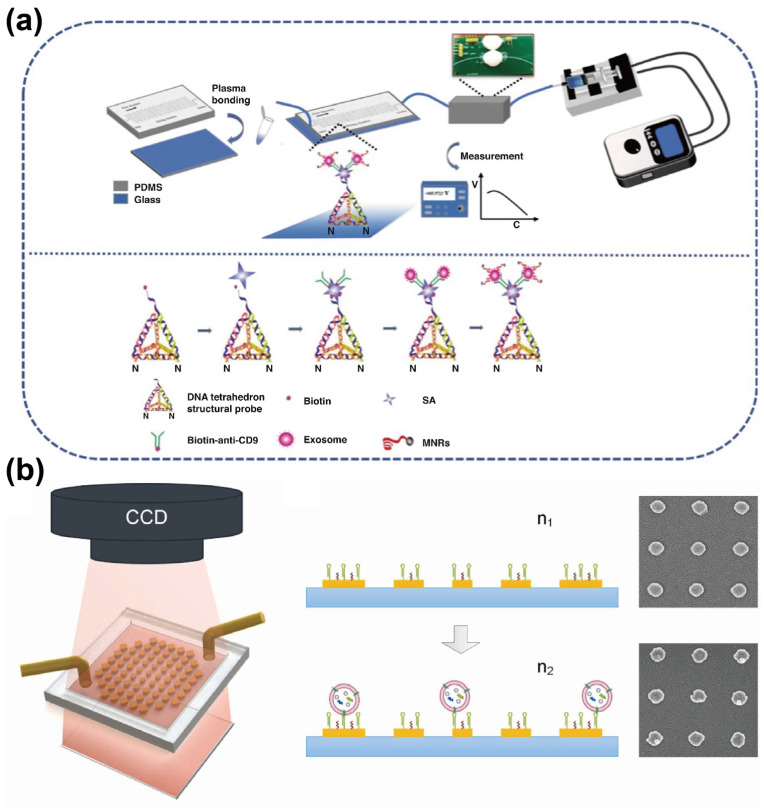
Schematic diagrams of other microfluidic–based aptasensors. (**a**) Schematic of the μFMS system and the exosome capture process. Reprinted from Ref. [60] under Creative Commons Attribution (CC BY) 4.0 License. https://creativecommons.org/licenses/by/4.0/ (accessed on 7 November 2022); (**b**) Schematic of a CGN aptasensor. Reprinted with permission from Ref. [63]. Copyright 2023 Elsevier B.V.

In addition to the aforementioned aptasensors, magnetic biosensors have also been proposed as effective tools for detecting exosomes, cells, and other targets. Generally, magnetic sensors involve the use of magnetic nanomaterials (MNPs) and associated labeling strategies [59]. These labeling strategies typically rely on affinity ligands to functionalize MNPs, thereby enabling the magnetic detection of target analytes. As previously discussed, owing to their high affinity and specificity, aptamers are often immobilized on MNPs, serving not only as capture probes for exosomes but as detection probes. Recently, Qian et al. [60] developed a DNA tetrahedral-structured probe (TSP)-mediated microfluidic magnetic detection system (μFMS). In this system, DNA TSPs immobilized on a chip are used to capture exosomes derived from human glioblastoma cells, while CD63 aptamer-functionalized MNPs act as magnetic nano-reporter probes, converting the captured exosomes into a detectable magnetic signal (Figure 9a). The magnetic signal is then measured using a magnetic detector equipped with an induction coil and a differential amplification circuit, effectively eliminating background noise. In PBS, the μFMS system achieved an LOD of 1.98 × 10^3^ particles/mL within an hour, with comparable performance in simulated serum samples, thus providing a low background interference and stable signal detection strategy for exosome analysis.

On the other hand, surface plasmon resonance (SPR)-based biosensors offer a label-free detection strategy by sensing local refractive index changes mediated by the binding of analytes to the sensing surface, resulting in a shift in optical resonance. With advantages such as high sensitivity, real-time analysis, and the potential for miniaturization, SPR is regarded as a powerful tool for label-free detection [61,62]. However, traditional SPR methods often require large and expensive equipment to measure resonance shifts, limiting their application as portable biosensors. To address this limitation, Feng et al. [63] developed an imaging-based concentric gradient nanoplasmonic (CGN) microfluidic sensor for the detection of tumor-derived exosomes. As shown in Figure 9b, the CGN sensor employs wafer-scale gradient plasmonic nanostructures that translate resonance wavelength shifts into centimeter-scale transmission pattern changes, thereby eliminating the dependence on costly, high-resolution spectrometers. Functionalizing the nanostructure arrays with CD63 aptamers allows the sensor to detect dynamic exosome binding specifically. The pattern movement is recorded via a CCD camera, enabling the aptasensor to achieve label-free and quantitative detection of cancer-derived exosomes with an LOD of 143 fM and a sensing performance as high as 9.23 × 10^−5^ RIU. The simplicity, robustness, and capability for large-area real-time imaging make the system a promising candidate for POCT applications.

The characteristics and inherent advantages and drawbacks of the aptasensors mentioned above are summarized in Table 1. While these aptasensors have demonstrated promising performance for exosome analysis, their application is still in the early stages. Therefore, additional studies using clinical samples are needed to confirm their accuracy in detecting complex, low-concentration analytes and to evaluate their reproducibility and stability under systematic testing.

## 5. Conclusions and Future Perspectives

Exosome-based liquid biopsy technologies provide a non-invasive, sensitive, and dynamic approach to cancer diagnosis. Compared with traditional analytical methods, aptamer-based microfluidic devices offer distinct advantages by combining the stability, high compatibility, and precise analyte targeting of aptamers with the integration, automation, and reduced reagent consumption of microfluidic systems. The integration of these elements has markedly improved the efficacy of exosome analysis. Recent advancements in aptamer-based microfluidic platforms have highlighted their potential for the isolation, amplification, and detection of exosomes. By targeting specific biomarkers on exosomes, these platforms have improved the efficiency of separation, purity, and recovery in exosome capture. They have also optimized signal amplification strategies and enhanced detection sensitivity and specificity through various signal detection techniques. Furthermore, some studies have tackled the challenge of exosomal heterogeneity by integrating multi-target detection, dual-modal detection, and exosome phenotyping into their designs. Despite all these achievements, several limitations still need to be addressed.

Firstly, although aptamers offer high specificity for recognizing and capturing exosomes, their binding stability can be disrupted by fluid shear forces or nuclease degradation in complex biological environments. Such conditions can further lead to a reduced binding affinity of aptamers and heighten the chances of non-specific interactions. Moreover, the limited availability of suitable aptamers for exosome detection continues to pose a significant challenge. Therefore, future efforts should focus on expanding the aptamer repertoire through SELEX technology to enhance their affinity, stability, and specificity. Additionally, affinity reagents that facilitate aptamer-target binding under physiological conditions should be developed.

Secondly, current aptamer-based microfluidic devices face challenges such as non-specific adsorption of target biomolecules to the walls of microfluidic channels, which can result in false-positive outcomes. Additionally, the intricate design of these systems often requires labor-intensive modification steps, further limiting their practical utility. To address these limitations, future research could focus on developing integrated multiplex detection systems. By leveraging multiple microchambers or synchronized signal amplification for various targets, such systems could streamline operations and simplify chip design without compromising detection sensitivity. Moreover, enhancing mass transfer within the chip through advanced structural design and precise fluid manipulation could significantly increase the collision between targets and recognition elements, thereby improving recognition efficiency.

Indeed, the aforementioned exosome detection strategies often involve lengthy detection times, extensive manual intervention, the need for sample pre-treatment, and the requirement of large, costly downstream detection equipment, all of which present substantial barriers to their practical application. To address these challenges, future research should focus on optimizing system stability and reproducibility while advancing cost-effective solutions and streamlining workflows. Furthermore, given the increasing demand for simplicity and portability in cancer diagnostics, the development of integrated “sample-in, answer-out” platforms tailored for POCT will likely become a key research focus in the near future.

## Figures and Tables

**Table 1:** Overview of the characteristics of recently reported aptamer-based microfluidic sensors for exosome detection.

Types of Sensors	Aptamer	Injected Samples	LOD (Particles/mL)	Cost of Time	Advantages	Potential Limitations	Refs.
Fluorescence	CD63	Cell culture medium; human serum samples	8.9 × 10^3^	119 min	High sensitivity; low sample consumption	Limited specificity; time-consuming	[43]
Fluorescence	PD-L1	Cell culture medium; human blood samples	1.58 × 10^5^	8 min (isolation); 2 h 25 min (detection)	One-step isolation	Complex chip design; high sample consumption	[44]
Fluorescence	EpCAM; CD63; PD-L1; PSMA; CA125; Nucleolin; PTK-7	Exosome from HepG2, MCF-7, HeLa and HEK-293T cell lines; human serum samples	8.69 × 10^3^	90 min	Superior capture performance	False-positive interference; pre-treatment required	[45]
Fluorescence	CD63	Exosome from A375, HepG2 cell lines and human plasma samples	6.89 × 10^3^	20 min	High accuracy; rapid detection	Pre-treatment required; high sample consumption	[47]
Electrochemistry	CD63	Exosome from HepG2 cell line	1 × 10^6^	15 min	Small sample volume; easy readout	Non-specific target capture; limited sensitivity	[48]
Electrochemistry	CD63	Exosome from HepG2 cell line; purified human serum samples	4.39 × 10^3^	<3.5 h	Label free; small sample volume	Time-consuming; pre-treatment required	[49]
Electrochemistry	EpCAM	Exosome from H1975 cell line and human serum samples	1.4 × 10^4^	60 min	Label free; low sample consumption; Simple operation	Low specificity; pre-treatment required	[51]
Electrochemistry	EpCAM	Exosome from MCF-7 cell line and human plasma samples	1.7 × 10^4^	<1 h	Superior electrochemical properties; low sample consumption	Limited specificity; complex chip design	[28]
SERS	CD63	Exosome from MCF-7 cell line	1 pmol/L (miRNA)	>3 h	High sensitivity; highly integrated	Limited specificity; time-consuming; complicated system	[35]
Fluorescence SERS	CD63	ExoStd human urine exosome standard sample; Exosome from plasma samples	1.3 × 10^6^	40 min (Immunofluorescent assay) 30 min (SERS assay)	Dual-mode detection; low sample consumption	Cumbersome off-chip modification; high-cost	[56]
SERS	HER2	Exosome from SKBR3 cell line and human plasma samples	4.5 log_10_	5 min per sample	Rapid response; “sample in, answer out” analysis; high throughput	Background interference; poor reproducibility	[57]
Magnetism	CD63	Exosome from U251 cell line; simulated serum samples	1.98 × 10^3^	≈1 h	Low background interference	Aptamer non-specific adsorption to MNPs	[60]
Nanoplasmonic	CD63	Exosome from A594 and MCF-7 cell lines and human plasma samples	143 fM	30 min	Label free; high sensitivity	Sensing depth constraints; limited specificity	[63]

## Abbreviations

The following abbreviations are used in this manuscript:

HCR	Hybridization chain reaction
RCA	Rolling circle amplification
SERS	Surface-enhanced Raman scattering
EVs	Extracellular vesicles
MBs	Magnetic beads
ssDNA	single-stranded DNA
SELEX	Systematic evolution of ligands by exponential enrichment
3D	Three-dimensional
IMB	Immunomagnetic bead
CCM	Carbon-coated magnetic
AFCSBs	Aptamer-functionalized core-shell barcodes
dsDNA	double-stranded DNA
LOD	Limit of detection
MNs	Magnetic nanospheres
TSA	Tyramine signal amplification
2D	Two-dimensional
PC	photonic crystal
SA-HRP	Streptavidin-horseradish peroxidase
DEIC	Dean-flow-coupled elasto-inertial microfluidic chip
LDA	Linear discriminant analysis
NTA	Nanoparticle tracking analysis
4-NTP	4-nitrothiophenol
POCT	Point-of-care testing
GNPs	Gold nanoparticles
MNPs	Magnetic nanomaterials
TSP	Tetrahedral-structured probe
SPR	Surface plasmon resonance
CGN	Concentric gradient nanoplasmonic
